# Chemokines and their receptors in oral squamous cell carcinoma: mechanisms, clinical significance, and therapeutic implications

**DOI:** 10.3389/fimmu.2025.1689695

**Published:** 2025-12-09

**Authors:** Dandan Li, Shuangshuang Chen, Jiahao Ji, Zhenwu Wang, Han Zhou, Ziyi Liao, Qun Tang

**Affiliations:** Medical School, Hunan University of Chinese Medicine, Changsha, China

**Keywords:** chemokines, chemokine receptors, oral squamous cell carcinoma, TME, immunology

## Abstract

Chemokines, a category of cytokines exhibiting chemotactic properties, have been thoroughly investigated as targets in cancer treatment in recent decades. In oral squamous cell carcinoma (OSCC), a prevalent and high-risk malignancy of the head and neck, chemokines interact with their specific receptors to initiate downstream signaling pathways. This signaling influences not only tumor cell proliferation, migration, invasion, and metastasis of oral cancer cells but also angiogenesis and vascular maturation. Furthermore, they modulate the tumor microenvironment (TME), which consists of diverse cellular and molecular components that collectively create a permissive niche for tumor growth, metastasis, and therapeutic resistance. This modulation impacts the recruitment and functionality of immune cells, which in turn influences tumor immune evasion and immune surveillance. Various chemokines and their receptors have distinct expression patterns in oral cancer tissues compared to normal tissues. Certain chemokines may function as prospective diagnostic markers, prognostic indicators, and therapeutic targets. In this review, we systematically summarize research advancements on chemokines in OSCC, elucidating their molecular mechanisms in tumor initiation and progression with a focus on the dualistic roles of key chemokine families (e.g., CCL2/5/20/19/21, CXCL1/8/12, CX3CL1) in regulating immune responses, tumor-stroma interactions, vascular remodeling, and chemotherapy resistance. We also recap current chemokine/receptor-targeted therapeutic strategies and discuss the limitations of existing research, including incomplete mechanistic understanding of understudied chemokine subfamilies (beyond CXC and CC subfamilies) and limited clinical translation of chemokine-based diagnostics and therapeutics. Finally, we propose future research directions: prioritizing patient stratification based on chemokine profiles, developing targeted delivery systems for chemokine antagonists, and combining these approaches with emerging therapies to overcome treatment resistance. This review underscores the critical role of chemokines in OSCC biology and their promising potential to guide the development of novel, effective therapeutic interventions.

## 1 Introduction

Chemokines are a family of small secreted proteins (6–14 kDa), typically comprising 70–125 amino acids, which share structural homology. They mediate directed cell migration by binding to specific G protein-coupled receptors (GPCRs) (1). Based on the arrangement of N-terminal cysteine residues, chemokines are classified into four subfamilies: CC, CXC, CX3C, and XC (2). To date, approximately 20 chemokine receptors have been identified (with a few additional candidates reported), all characterized by seven transmembrane domains connected by extracellular and intracellular loops. These receptors signal primarily through heterotrimeric Gi proteins to exert their biological functions (3).

Initially recognized for their role in directing immune cell migration, chemokines primarily regulate the trafficking of leukocytes such as monocytes, eosinophils, and dendritic cells to inflammatory sites (4–8). Emerging evidence indicates that chemokines also directly influence non-immune cells, including tumor cells and vascular endothelial cells. Within the TME, this interaction contributes to the regulation of tumor cell proliferation, invasion, and metastasis (9, 10).

The CC subfamily, characterized by adjacent N-terminal cysteine residues (Cys-Cys motif), represents the largest (28 members) and most diverse group of chemokines. These chemokines interact with extracellular matrix components to coordinate the directed migration of immune cells—including monocytes, macrophages, lymphocytes, and certain neutrophils (11, 12). Accumulating evidence underscores their pivotal roles in fibrosis, aging, and associated pathologies (13, 14).

CXC chemokines feature a characteristic Cys-X-Cys motif (where X denotes any amino acid). Like the CC subfamily, CXC chemokines exhibit dual roles in tumor biology—both promoting and inhibiting tumor progression, angiogenesis, metastasis, and tumor immunity (15–17). Particular research focus has centered on CXCL9-11, CXCL4, and its variant CXCL4L1, which signal through the CXCR3 subtype. Notably, the two subtypes of CXCR3- CXCR3-A and CXCR3-B - play completely opposite roles in tumor progression: CXCR3-A promotes tumor growth, while CXCR3-B inhibits tumor development. Their regulatory mechanisms not only involve direct impacts on tumor cells, but also indirectly affect tumor progression by regulating angiogenesis and the TME (18).

The CX3C subfamily is exclusively represented by CX3CL1 (fractalkine), featuring a Cys-X-X-X-Cys motif. This chemokine exists in both membrane-anchored and soluble proteolytic forms, promoting the chemotaxis of monocytes, T cell, and natural killer cell(NK cell) while mediating cellular adhesion through its receptor CX3CR1—including critical processes like neuron-microglia communication (19).

The XC subfamily, defined by a single N-terminal cysteine (Cys), comprises XCL1 (lymphotactin-α, LT-α) and XCL2 (lymphotactin-β, LT-β). These chemokines specifically regulate the migration of T cells and NK cells through interaction with XCR1, particularly within thymic and lymph node microenvironments (20–22).

In the TME, chemokines critically influence tumor progression by regulating immune cell infiltration, angiogenesis, tumor cell proliferation, and metastasis (23). In OSCC, they modulate tumor progression through immune cell recruitment, angiogenesis, matrix remodeling, and fibrosis. Studies have shown that CCR6/CCL20 drives proliferation and migration in lung adenocarcinoma via autocrine/paracrine mechanisms (24, 25), while the CXCL12/CXCR4 axis promotes OSCC invasion and distant metastasis—with CXCR4 blockade reducing lymph node metastasis (26, 27). In tongue squamous cell carcinoma (TSCC), CXCL9/CXCR3 activates Akt signaling pathway, inducing epithelial-mesenchymal transition (EMT) and cytoskeletal rearrangement to facilitate invasion and metastasis (28). Notably, CX3CL1 may play a dual role in promoting and inhibiting tumors. Studies have shown that CX3CL1-CX3CR1 signaling transduction activates transcription factors SATB1 and BTG2, thereby promoting the differentiation of TCF7 Treg cells into functionally enhanced TNFRSF9 Treg cells (29). Conversely, IL-15RαTAMs can reduce the protein level of the chemokine CX3CL1 in tumor cells by releasing the IL-15/IL-15Rα complex (IL-15Rc), inhibiting the recruitment of CD8+T cells (30). Beyond their core functions in immune recruitment and inflammation, chemokines also govern physiological processes including embryonic development, angiogenesis, and tissue homeostasis.

The investigation of chemokine functions holds significant theoretical and clinical relevance. At the fundamental research level, elucidating the specific mechanisms of chemokines may yield novel therapeutic targets for cancer treatment. In clinical diagnostics, specific chemokines (e.g., CXCL8 and CCL2) serve as valuable biomarkers, with their expression levels demonstrating significant correlations with tumor stage and prognosis. Therapeutically, clinical trials targeting chemokine receptors (e.g., the CXCR4 antagonist Plerixafor) have shown promise in suppressing metastasis, while combination strategies with immune checkpoint inhibitors (e.g., anti-PD-1 antibodies) significantly enhance treatment efficacy. Furthermore, chemokine profiling provides new insights for molecular subtyping, prognosis assessment, and recurrence monitoring in oncology. However, the dual-functional nature (“double-edged sword” effect) of chemokines and persistent translational barriers warrant further mechanistic investigation and therapeutic breakthroughs.

In this comprehensive review, we systematically summarize the current understanding of the chemokine/chemokine receptor network in OSCC. We critically examine mechanisms through which chemokines remodel the TME and evaluate their clinical translation potential. Specifically, we evaluate: the diagnostic and prognostic value of chemokine profiles in OSCC, as well as emerging therapeutic strategies targeting chemokine pathways. Additionally, we emphasize the existing challenges and future research directions in this field. This review consolidates contemporary perspectives on the multifaceted roles of chemokines in OSCC pathogenesis while underscoring their translational promise for therapeutic development.

## 2 Chemokine/chemokine receptors in OSCC

Oral cancer ranks among the most prevalent malignant tumors of the head and neck, with OSCC accounting for over 90% of cases. OSCC is characterized by its insidious onset, high mortality rate, and rising global incidence. In 2020, the Global Cancer Observatory reported approximately 377,713 new cases of OSCC worldwide (31). The five-year global prevalence of OSCC reached nearly one million cases (959,248), particularly affecting high-risk groups including smokers, alcohol consumers, individuals with human papillomavirus (HPV) infections, and chronic betel nut chewers (32). This disease imposes a substantial global public health burden and represents a critical health challenge. OSCC exhibits aggressive behavior with a high propensity for metastasis, often causing local tissue destruction, functional impairment, and dissemination to distant organs (33). Lymph node metastasis is a primary contributor to poor prognosis, severely threatening patient survival and quality of life. Once metastasis occurs, the five-year survival rate typically falls below 50% (34). Current OSCC management remains primarily surgical, supplemented with radiotherapy and chemotherapy. Despite advances in multimodal therapies, the 5-year survival rate has not significantly improved over recent decades. This stagnation largely stems from challenges in early diagnosis, high rates of local recurrence, and frequent distant metastases (35).

In recent years, the TME has emerged as a major focus of scientific research, with its critical role in the initiation, development, and progression of OSCC now widely recognized (36). The OSCC TME comprises both cellular and stromal components. Diverse cell populations in the TME, including tumor-associated macrophages (TAMs), cancer-associated fibroblasts (CAFs), T lymphocytes, tumor-associated neutrophils (TANs), and dendritic cells (DCs), form a complex and vital cellular network. OSCC cells leverage this network by secreting RNAs, proteins, cytokines, and metabolites to regulate processes such as proliferation, invasion, migration, and angiogenesis. Chemokines, small cytokines with chemotactic activity, play a particularly important role. They orchestrate the precise migration and spatial distribution of immune cell subpopulations through G protein-coupled receptor-mediated signaling cascades. During OSCC evolution, the chemokine-receptor system profoundly influences the dynamic equilibrium of the TME. This system modulates inflammatory responses, immune surveillance, and tumor progression by directing the targeted migration of immune and other cells(**Table 1**). Aberrant expression of numerous chemokines and their receptors in OSCC has been directly linked to key tumor biological behaviors, including cell proliferation, migration, invasion, and immune evasion. The role of chemokines in OSCC is dual, exhibiting both tumor-promoting and tumor-suppressive functions. Consequently, in-depth exploration of chemokine mechanisms in OSCC holds significant promise. Such research will not only advance our understanding of OSCC pathogenesis but also potentially provide a theoretical foundation for developing novel diagnostic markers and therapeutic strategies.

**Table 1:** Chemokines and their receptors in oral squamous cell carcinoma.

Chemokines	Family category	Receptor	Sources	Function in OSCC
CCL2	CC	CCR2	Tumor cells (37), TAMs (38), endothelial cells (39), MC (40), TANs (41) ,fibroblasts, smooth muscle cells (39)	Promote the migration, invasion, and metastasis of tumor cells (37)(*In Vitro*); Promote angiogenesis (42)(*In Vitro)*; Mediate the interaction between mast cells (MC) and tumor cells (40)(*In Vitro*);Participate in the process of polarization of macrophages towards the M2 phenotype (43)(*In Vitro).*
CCL5	CC	CCR5	Tumor cells (44), macrophages (45),monocytes (46) , mast cells (46), T cells (46), endothelial cells (47)	Promote invasion and angiogenesis (44)(*In Vitro)*;Promote the polarization of M2 macrophages and form an immunosuppressive microenvironment (45)(*In Vivo)*;Promote regional lymph node metastasis (48)(*Ex Vivo)*.
CCL20	CC	CCR6	Tumor cells (49), T cells (50),CAFs (51), endothelial cells (52),DCs (52) ,natural killer cells (52)	Distinguish OSCC patients from healthy people (49)(*Ex Vivo)*;Recruit CCR6+ Treg cells (50)(*Ex Vivo)*; Promote the formation of the inflammatory microenvironment (53)(*In Vivo)*.
CCL19/CCL21	CC	CCR7	Tumor cells (54),TAMs (55), DCs (56),Lymphatic endothelial cells (56), fibroblasts (57), endothelial cells (58)	Construct immunosuppressive TME (59)(*In Vivo)*;Promote EMT and enhance the characteristics of tumor stem cells (60)(*In Vitro)*;Promote M2 polarization in THP-1-derived macrophages (55)(*In Vitro)*;Promote tumor growth (54)(*In Vivo)*.
CXCL1	CXC	CXCR1/CXCR2	Tumor cells (61), CAFs (62),TAMs (63),endothelial cells (64),MDSCs (65)	Activate tumor-associated macrophages (TAMs), induce M2 polarization and secrete epidermal growth factor (EGF) (63)(*In Vitro)*;Promote the transformation of normal fibroblasts (NOFs) into cancer-promoting cancer-associated fibroblasts (CAFs) (62)(*In Vitro)*;Promote tumor invasion and migration (61)(*In Vitro)*;Promote tumor recurrence (66)(*Ex Vivo)*.
CXCL8	CXC	CXCR1/CXCR2	Tumor cells (67), CAFs (68),TAMs (68), monocytes (69), macrophages (69), fibroblasts (69), endothelial cells (69)	Recruit mesenchymal stromal cells (e.g., BMSCs, DPSCs) to drive tumor progression (67)(*In Vitro)*;Predict tumor recurrence and occult lymph node metastasis (70)(*Ex Vivo)*.
CXCL12	CXC	CXCR4/CXCR7	Tumor cells (26), CAFs (71),TAMs (26),stromal cells (72) ,fibroblasts (72) , dendritic cells (72)	Promote cancer stem cell (CSC)-like cell formation (26)(*In Vitro)*; Promote the migration and invasion of tumor cells and participate in chemotherapy-induced EMT (73)(*Ex Vivo)*;Aggregate regulatory T cells (FoxP3+ T cells) in tumor tissues; Establish communication with CD4+/CD8+ T cells and B cells, regulate lymphocyte recruitment and anti - tumor immunity (74)(*Ex Vivo)*.
CX3CL1	CX3C	CX3CR1	Tumor cells (75), CAFs (68), TAMs (68), NK cells (76), T cells (76),Macrophages (77), DCs (77), smooth muscle cells (77), and neurons (77)	Promote the metastasis of cervical lymph nodes (LN) and increase the formation of tumor vascular-like structures (VLS) (78)(*In Vivo)*;Induce the migration of tumor cells (75)(*In Vitro)*;Reverse the cancer-promoting effect of lipopolysaccharide (LPS) partially (79)(*In Vitro)*;Recruit effector cells of CD57+ lymphocytes into the tumor tissue (80)(*Ex Vivo)*.

### 2.1 CCL2

CCL2, also known as monocyte chemoattractant protein-1 (MCP-1), is a 13 kDa protein composed of 76 amino acids. Its encoding gene resides on chromosome 17q11.2 (81). CCL2 typically binds its receptor, CCR2 (82). The CCL2-CCR2 axis plays significant roles in the development and progression of various cancers, including OSCC. CCL2 recruits monocytes, memory T lymphocytes, and NK cells, and is expressed by diverse cell types such as epithelial, endothelial, myeloid, fibroblast, and smooth muscle cells. Within the OSCC microenvironment, this axis contributes to angiogenesis, tumorigenesis, metastasis, and prognosis.

Elevated CCL2 expression is strongly associated with OSCC progression and prognosis. Serum CCL2 levels serve as a potential tumor biomarker and correlate with disease advancement. Furthermore, CCL2 levels represent a useful prognostic parameter in OSCC patients (83). By performing immunohistochemical experiments on 41 patient samples undergoing neck dissection, Fujita et al. revealed that TANs and SCC parenchyma at the primary tumor site produced CCL2. Their findings suggest the CCL2-CCR2 axis facilitates lymphatic metastasis by mediating interactions between the primary tumor site and marginal sinus histiocytosis in regional lymph nodes (84).

Multiple evidences now implicate CCL2 in the migration and invasion of OSCC. EMT, a reversible process crucial for early metastasis, involves polarized epithelial cells losing their basal membrane attachment and undergoing biochemical changes that enhance migratory and invasive capabilities. Studies demonstrate that recombinant CCL2 (rCCL2) induces EMT in OSCC cells by up-regulating Snail, characterized by reduced E-cadherin and increased vimentin expression. Significantly, CCL2-neutralizing antibodies block this CCL2-induced EMT (37). Notably, recent research reveals a novel mechanism for the natural flavonoid Oroxylin A (Oro-A): it exerts non-cytotoxic anti-metastatic effects by targeting the CCL2 pathway. In OSCC models, long-term Oro-A exposure significantly inhibits both CCL2 mRNA transcription and protein synthesis. Consequently, Oro-A blocks activation of the downstream signaling molecules phosphorylated ERK1/2 (p-ERK1/2) and NF-κB, leading to reduced expression of the matrix metalloproteinase-2 (MMP2)​ and matrix metalloproteinase-9 (MMP9) (85). These findings collectively suggest that Oro-A may inhibit OSCC invasion and metastasis by disrupting multi-target signaling networks mediated by CCL2, including pathways associated with EMT.

CCL2 also critically modulates diverse immune cell behaviors within the OSCC TME. A study conducted MC/OSCC co-culture systems detected significantly increased CCL2 release, indicating that CCL2 may be a mediator of MC/OSCC interaction, and CCL2 may be involved in MC recruitment (40). Furthermore, recent research investigating MC-tumor cell interaction-related genes and microRNA expression profiles underscores the clinical relevance of CCL2 signaling and the differential expression of the CCL2/CCR2 axis in OSCC (86). In addition, the immunosuppressive effect of CCL2 is also related to the polarization of M2 macrophages. Studies demonstrate that KIF4A, a member of the kinesin superfamily (KIF), regulates both macrophage migration and their polarization towards the M2 phenotype. Significantly, KIF4A achieves both functions by modulating CCL2 expression (43).

Tumor angiogenesis plays a crucial role in tumor growth, maintenance and metastasis, and CCL2/CCR2 axis plays a key role in the angiogenesis of OSCC. Mechanistically, the proteasome activator PA28γ (REGγ) promotes tumor cell secretion of IL-6 and CCL2 by activating the p-NF-κB pathway. These cytokines subsequently stimulate endothelial cell IL-6 receptor and CCR2 signaling, thereby driving neovascularization (87). Furthermore, *in vivo* and *in vitro* studies demonstrate that elevated expression levels of MCP-1 and vascular endothelial growth factor A (VEGF-A) positively correlate with OSCC disease stage. CCL2 directly increases VEGF-A expression, promoting angiogenesis via the CCR2/ILK/MEK1/2 signaling pathway (42).

### 2.2 CCL5

CCL5, also known as RANTES (regulated upon activation, expressed and secreted by normal T cells), plays a key role in the immune response by attracting immune cells including T cells, eosinophils, and basophils to the site of inflammation (88). Elevated CCL5 expression is observed in various tumors and correlates with enhanced tumor growth and poor prognosis. Pan-cancer studies highlight its therapeutic potential in malignancies such as renal cell carcinoma (KIRC) and esophageal carcinoma (ESCA) (89). Its primary receptor, CCR5, is expressed on macrophages, activated T cells, NK cells, and endothelial progenitors. CCR5 upregulates pro-inflammatory responses by modulating immune cell behavior, survival, and tissue retention (90).

Microbial infections are closely associated with the development of OSCC, and CCL5 expression may be induced in this process. Fusobacterium nucleatum infection can significantly upregulate CCL5 expression in malignant oral keratinocytes (MOKs) (e.g., H357, H376) by activating their transcriptional response. This elevated CCL5 induces MMP9 and VEGF-A, promoting tumor invasion and angiogenesis (44). In contrast, Porphyromonas gingivalis (Pg) infection accelerates OSCC progression by up-regulating CCL5 in TAMs, which promotes the polarization of M2-type macrophages (M2 macrophages) and creates an immunosuppressive microenvironment (45).

Overexpression of CCR5/CCL5 axis proteins in OSCC is associated with poor outcomes, including regional lymph node metastasis and reduced overall survival, signifying a highly aggressive tumor phenotype and unfavorable prognosis (48). This association likely stems from CCL5’s role in orchestrating the leukocyte population within the tumor. By binding CCR5, CCL5 mediates the infiltration of TAMs and suppresses the activity of potential antitumor T cells, thereby altering the TME to promote disease progression (91).

OSCC exhibits a complex molecular regulatory network. Long-chain non-coding RNA ZFAS1 accelerates OSCC progression by adsorbing miR-6499-3p (a target miRNA of CCL5), deregulating the inhibitory effect on CCL5 and promoting its expression, which in turn activates the CCL5-CCL5 signaling axis (92). Furthermore, the interaction between the Axin2-Snail axis and CCL5 significantly contributes to OSCC pathogenesis and represents a potential therapeutic target. Studies demonstrate that activation of the Axin2-Snail axis increases CCL5 expression, which subsequently modulates CAF function and influences other stromal components, ultimately promoting tumor invasion and metastasis (93).

### 2.3 CCL20

CCL20, a member of the CC chemokine subfamily, is also known as macrophage inflammatory protein 3 alpha (MIP3 alpha) or liver and activation-regulated chemokines (LARC). It was first identified and characterized in hepatocytes (94). CCR6 is the only known high-affinity homologous receptor for CCL20, which is expressed predominantly on immature DCs, innate lymphoid cells (ILCs), regulatory D4+ T cells (Tregs), Th17 cells, and B cells. The CCL20-CCR6 axis represents a well-established therapeutic target in autoimmune disorders including inflammatory bowel disease (IBD), psoriasis (PS), and rheumatoid arthritis (RA) (95). Emerging evidence indicates that CCL20 signaling also critically influences the TME of multiple malignancies, including OSCC (96).

CCL20 exerts multifaceted critical functions in the OSCC microenvironment. Studies demonstrate markedly elevated CCL20 expression in both OSCC tissues and patient saliva. Notably, *Fusobacterium*, as a dominant bacterium in OSCC, is closely associated with CCL20 expression levels. Salivary CCL20 quantification shows promise as a non-invasive biomarker, effectively distinguishing OSCC patients from healthy volunteers (HVs) (49). Furthermore, immunohistochemical analysis by Mandal et al. first proposed that Fusobacterium nucleatum-positive OSCC tumorigenesis may involve CCL20 dysregulation, particularly within the Indian cohort. This finding deepens our mechanistic understanding of Fusobacterium-mediated oral carcinogenesis (97).

Tregs are abundant in tumor tissues and suppress effective anti-tumor immunity. However, the mechanisms driving Treg infiltration into tumors and their precise impact on cancer progression remain incompletely understood. In OSCC, CCL20 favors the recruitment and retention of CCR6+ Treg cells through its interaction with the CCR6 receptor. It has been shown that during local inflammation, effector/memory-like CCR6+ Treg cells may be rapidly attracted to tissues to constrain excessive inflammation. Notably, CCR6+ Treg cells were enriched in tumor-infiltrating lymphocytes (TILs) and metastatic lymph nodes compared to peripheral blood mononuclear cells (PBMC) (50). Further studies revealed that P. gingivalis infection with OSCC resulted in a reduction of CD8+ T cells in the TME. This was attributed to the recruitment of CCR6+ Treg cells by P. gingivalis through tumor cells, thereby reducing the proportion of effector CD8+ T cells in tumor-infiltrating lymphocytes and inhibiting their effector function, which in turn formed an immunosuppressive microenvironment in the TME and promoted the development of the malignant phenotype of OSCC (53).

Collectively, CCL20 critically shapes the inflammatory landscape, orchestrates immune cell recruitment/regulation, and mediates bacterium-tumor crosstalk in OSCC. Elucidating its mechanisms will advance diagnostic biomarker development and therapeutic strategies for OSCC.

### 2.4 CCL19/CCL21

CCL19 and CCL21, also known as EBI1-ligand chemokine/macrophage inflammatory protein-3β (ELC/MIP-3β) and secondary lymphoid tissue chemokine (SLC), are the major chemokines expressed primarily in secondary lymphoid tissues (98, 99). Their expression is upregulated in a variety of diseases, including atherosclerosis (100), cancer (82), bone disease (101), and asthma (102). CCL21 contains a C-terminus that is 32 amino acids longer than CCL19, conferring unique binding properties. Both bind to the CCR7 receptor and their biological functions are primarily mediated through CCR7. CCR7 is widely expressed on diverse immune cells and certain tumor cells, serving as a critical regulator of lymphocyte migration to secondary lymphoid organs (103). The CCL19/CCL21-CCR7 axis plays an important role in malignancies such as OSCC.

In OSCC, the CCL19 and CCL21 signaling pathways have an important impact on the TME and immune response by interacting with the CCR7 receptor. Recent studies reveal that the extracellular matrix protein tenascin-C (TNC) remodels the CCL21/CCR7 signaling network to foster immunosuppressive TMEs. Specifically, TNC upregulates the expression level of CCL21 in fibroblastic reticulocytes (FRCs) and lymphatic endothelial cells (LECs). By altering the chemokine concentration gradient between the lymph node-tumor, it leads to impaired DCs migration and defective cytotoxic T-lymphocyte (CTL) activation (59). Notably, this signaling axis also activates the JAK2/STAT3 pathway, which synergistically promotes EMT and enhances tumor stem cell properties (60). Furthermore, the proportion of FRCs expressing high levels of CCL19 was significantly increased in primary tumors after neoadjuvant chemoimmunotherapy (NACI) treatment, whereas the proportion of these cells was reduced in metastatic lymph nodes of patients in the no-pathological-response (NPR) group, suggesting that CCL19+ FRCs may have a potential antitumor effect (104).

Tertiary lymphoid structures (TLS) are ectopic lymphoid organs that develop in non-lymphoid tissues. Their presence correlates strongly with favorable clinical outcomes and enhanced immunotherapy responses across multiple cancers. In OSCC, CCR7 has been identified as a key TLS-related gene (TLSRG). Studies demonstrate that elevated CCR7 expression associates with improved prognosis in OSCC patients. A risk score model incorporating CCR7 effectively stratifies patients into high- and low-risk groups, demonstrating predictive value for both overall survival (OS) and disease-free survival (DFS). Consequently, CCR7 expression levels represent a potential prognostic biomarker for favorable outcomes in OSCC (105).

However, in contrast, some studies suggest that CCR7 plays a pro-cancer role in OSCC. *In vitro* experiments confirmed that activation of CCR7 signaling by OSCC cells through secretion of CCL19/CCL21 promotes M2 polarization in THP-1-derived macrophages (55). Knockout studies further revealed that CCR7 deletion significantly inhibited tumor growth, and the mechanism may involve the blockage of M2 polarization due to the upregulation of Dusp1 expression (106). In addition, the proportion of CD8+ T cells was expressed higher in OSCC tumor tissues from CCR7-deficient mice, suggesting that CCR7 may promote tumor growth by inhibiting the activation of initial CD8+ T cells, thereby affecting the proliferation and replenishment of antitumor immune CD8+ T cells (54). Another study demonstrated that specific blockade of the CCR7 pathway by a specific protein hydrolysis-resistant TC6-D3 peptide significantly reduced lymph node tumor load, promoted primary tumor CD8+ T-cell infiltration, and enhanced lymph node anti-tumor immune response, which is expected to be a new option for the treatment of OSCC (107).

### 2.5 CXCL1

CXCL1 (C-X-C motif chemokine ligand 1), an important member of the CXC chemokine subfamily, exhibits pleiotropic functions in inflammatory responses and TME remodeling. Also known as neutrophil-activating peptide-3 (NAP-3), growth-regulated oncogene-α (GRO-α), or melanoma growth-stimulatory activity (MGSA) (108). CXCL1 primarily signals through the G protein-coupled receptors CXCR1 and CXCR2. Notably, CXCR2 serves as its dominant functional receptor under physiological conditions, while CXCR1 activation requires substantially higher ligand concentrations (62).

Recent studies have revealed that CXCL1 plays a key role in the remodeling of the microenvironment of OSCC. OSCC cell lines (e.g., Cal-27) demonstrate significantly elevated CXCL1 expression compared to immortalized keratinocytes (HaCaT). Mechanistically, tumor-derived CXCL1 activates TAMs via paracrine secretion, driving their M2 polarization and stimulating EGF secretion. This EGF subsequently induces EMT in Cal-27 cells and initiates the up-regulation of CXCL1 in a positive feedback manner. This CXCL1-EGF-NF-κB positive feedback axis drives OSCC progression and immune microenvironment remodeling (63).

CAFs, recognized as senescent cells, stimulate precancerous and malignant epithelial cell growth, driving cancer progression across multiple malignancies. In OSCC, CXCL1 induces the transformation of normal oral fibroblasts (NOFs) into senescent CAFs via an autocrine mechanism, thereby promoting tumor growth (62). Subsequent work by Wei et al. revealed that IL-1β secreted by primary tumor cells stimulates CAFs to produce CXCL1. This chemokine then binds CXCR2 receptors on cancer cells, inducing MMP1 production in the TME and enhancing cancer cell migration and invasion (61). Cancer dormancy plays a decisive role in the recurrence and metastasis of various cancers, and differentiated embryonic chondrocyte gene 2 (DEC2) is strongly correlated with mediating cancer dormancy. It was found that secretion of CXCL1 by CAFs also reactivated dormant OSCC cells by down-regulating DEC2 expression in tumor cells, and this process was significantly associated with the recurrence and infiltration of cancer-associated fibroblasts (66). Collectively, these studies demonstrate a tightly coupled interaction between CXCL1 and CAFs in OSCC, establishing this axis as a promising therapeutic target.

### 2.6 CXCL8

CXCL8, among the earliest discovered and most extensively studied pro-inflammatory chemokines, has remained a major research focus in inflammation and immunology since its initial identification (109). The signaling pathway of CXCL8 is centered on the precise binding of the ligand CXCL8 to the specific receptors CXCR1 and CXCR2, which plays an irreplaceable role in mediating neutrophil chemotaxis and activating the inflammatory cascade (110). In recent years, the exploration of the mechanism of CXCL8-CXCR1/2 signaling in the process of tumorigenesis and development has been deepening.

CXCL8 serves as a well-established biomarker in OSCC, demonstrating significant diagnostic and prognostic value (111). Multiple studies confirm substantial upregulation of CXCL8 gene expression in OSCC tissues compared to normal counterparts. Li et al. systematically analyzed head and neck squamous cell carcinoma (HNSCC) datasets, revealing consistently elevated CXCL8 expression in tumor samples (112).

León et al. further established CXCL8 as an effective predictive biomarker for recurrence risk in HNSCC patients (113). Given that CXCL8 is significantly associated with the prognosis of patients and plays an important role in predicting tumor recurrence and occult lymph node metastasis, it is expected to become a key molecular target to guide the formulation of individualized drug treatment strategies for high-risk patients with OSCC (70).

A bidirectional regulatory mechanism between cancer cells and mesenchymal cells within the TME is now well-documented. Taking bone marrow mesenchymal stem cells (BMSC) as an example, they show the characteristics of migrating to the tumor site in tumors such as hepatocellular carcinoma. Lin’s team confirmed through *in vivo* and *in vitro* experiments that BMSC can be recruited to the OSCC region and interact with OSCC cells to drive tumor progression via the CXCL8/CXCR2 and TGF-β/Ras/Raf/Erk signaling axes (67). Complementary studies reveal that up-regulated CPNE7 expression in mesenchymal stromal cells (MSCs) regulates CXCL8 secretion by activating the NF-κB pathway, thereby inducing EMT and promoting OSCC cell metastasis (114). Notably, CXCL8 secreted by OSCC cells acts as a chemoattractant for dental pulp mesenchymal stem cells (DPSCs) through CXCR2 receptors on DPSC surfaces. This recruitment mechanism provides the foundation for developing DPSC membrane-coated metal-organic framework nanoparticles as targeted OSCC therapeutics (115).

### 2.7 CXCL12

CXCL12, C-X-C motif chemokine ligand 12, also known as stromal cell-derived factor-1 (SDF-1), is a key member of the CXC subfamily of chemokines. This factor is encoded by several genes and can bind to specific receptors CXCR4 and CXCR7, activate downstream signaling pathways such as PI3K/AKT and RAS/MAPK, critically regulating both physiological and pathological processes (116). Under physiological conditions, CXCL12 mediates key processes including embryonic and neural development, hematopoietic stem cell (HSC) homing and retention in the bone marrow niche, and angiogenesis through regulation of vascular endothelial cell migration and proliferation (72, 110). Importantly, the CXCL12/CXCR4 axis has become an important drug target for tumor therapy due to its critical role in cancer stem cell maintenance (117).

CXCL12 plays a key role in OSCC. It is mainly secreted by CAFs, especially inflammatory CAFs (iCAFs) (71). It drives multiple oncogenic processes through CXCR4/CXCR7 receptor binding. The CXCL12/CXCR4 axis recruits monocytes, induces their differentiation into M2 macrophages, and promotes OSCC cell transformation into cancer stem cell (CSC)-like phenotypes, thereby accelerating tumor proliferation (26). Meanwhile, using spatial transcriptomics, Liu et al. revealed the association between metabolic heterogeneity and local immunosuppression in OSCC tumors. The experimental results showed that in the hypoxic hypermetabolic zone, CXCL12 secreted by iCAFs recruited Tregs to secrete TGF-β1 through the CXCL12-ACKR3 axis, forming an immunosuppressive microenvironment and contributing to tumor immune escape (118).

In addition, Moreover, CXCL12 influences disease progression and patient prognosis through multifaceted mechanisms. Studies confirm its expression in tumor, stroma, and muscle cells of primary tongue OSCC tissues. The CXCL12 expression level is negatively correlated with tumor grade, and its high expression is associated with improved prognosis and overall survival. Furthermore, interactions between lingual skeletal muscle cells and tumor cells appear to influence tumor progression (119). This suggests that the role of CXCL12 in OSCC is complex and multifaceted, which may be related to the role of CXCL12 in different tumor stages and different microenvironments. Immunohistochemical evaluation of CXCL12 and CXCR4 expression in specimens from oral potentially malignant disorders (OLK) and OSCC revealed that the CXCL12/CXCR4 axis plays a crucial role in oral carcinogenesis, with its abnormal activation persisting from the precancerous lesion stage to the development of overt malignancy (120). Another study demonstrated that exogenous CXCL12 synergizes with CXCR4 and CXCR7 to facilitate the migration and invasion of OSCC cells. Furthermore, CXCL12 participates in chemotherapy-induced EMT and regulates interactions between tumor cells and CAFs (73).

In the context of TME regulation, CXCL12 exhibits paradoxical yet integrated functions. On one hand, its high expression can induce the abnormal accumulation of regulatory T cells (FoxP3+ T cells) in tumor tissues, creating an immunosuppressive microenvironment (74). On the other hand, the activated fibroblastic reticular cell subset C13-CCL19+ FRCs following neoadjuvant chemotherapy (NACI) can establish extensive communication with CD4+ T cells, CD8+ T cells, and B cells through the CXCL12-CXCR4 axis, potentially playing a positive regulatory role in lymphocyte recruitment and anti-tumor immune responses (104). These findings highlight the dual value of CXCL12 as both a tumor progression regulator and clinical outcome predictor.

### 2.8 CX3CL1

CX3CL1, or Fractalkine, can exist both in soluble and membrane-bound forms, with the latter anchored to the cell surface to mediate cell adhesion and signaling. As the sole member of the CX3C motif chemokine family, plays a dual role in immunity and tumor progression due to its high affinity for the receptor CX3CR1 (76). On one hand, it can increase lymphocyte infiltration, including cytotoxic T cells, NK cells, and DCs. Furthermore, CX3CL1 expressed by DCs promotes tumor infiltration of CD4+ and CD8+ T cells, correlating with improved prognosis (121). On the other hand, the CX3CL1/CX3CR1 axis promotes cancer cell proliferation, migration, and invasion, recruits pro-tumor immune cells, and promotes tumor angiogenesis through TAMs (122).

In the study of syngeneic mouse OSCC cell lines with different characteristics, MOC1 (non-metastatic, indolent) and MOC2 (invasive, metastatic), *in vitro* experiments showed that CX3CL1 overexpression inhibited cell proliferation and promoted cell migration in both MOC1 and MOC2 cells, and CX3CR1+ cells were recruited to the TME (TME) of MOC tumors after CX3CL1 overexpression. However, *in vivo*, its effects showed significant differences. In MOC1 tumors, the increase of CX3CL1 led to a smaller tumor size and increased tumor keratinization, indicating its potential anti-tumor effect. In MOC2 tumors, CX3CL1 promoted the metastasis of cervical lymph nodes (LNs), increased the formation of tumor vascular-like structures (VLS), and significantly increased the invasion of tumor cells within VLS, indicating its pro-metastatic effect. Moreover, the enrichment of CX3CL1 in metastatic lymph nodes may serve as a potential marker for poor prognosis in OSCC (78). In addition, studies have shown that CX3CL1 plays a crucial role in regulating the cell adhesion, migration, and survival of human cancer cells. In OSCC cells, the cell movement induced by CX3CL1 is closely related to the upregulation of intercellular adhesion molecule-1 (ICAM-1) expression. Further research found that CX3CL1 promotes ICAM-1 expression through the CX3CR1 and PLCβ/PKCα/c-Src signaling pathways, and then induces the migration of OSCC cells (75). This indicates that the CX3CL1-CX3CR1-mediated signaling pathway is closely associated with tumor movement and plays a key role in the occurrence and development of human OSCC. It is expected to be an important indicator for predicting the prognosis of human OSCC. In highly invasive OSCC cell lines, the mRNA expression of CX3CL1 is upregulated. CX3CL1 can induce the migration of oral cancer cells in a dose-dependent manner, and CX3CL1-positive nerves can attract CX3CR1-positive tumor cells, thereby promoting the development of perineural invasion (PNI) in OSCC patients (123).

However, emerging evidence from several studies paradoxically suggests that CX3CL1 may exhibit tumor-suppressive effects in OSCC, contradicting its established pro-tumorigenic role. This highlights the complex and context-dependent nature of CX3CL1’s function in cancer biology. It has been confirmed that both CX3CL1 and its receptor CX3CR1 exhibit low expression in OSCC, while soluble CX3CL1 (sCX3CL1) is highly expressed. Overexpression of CX3CL1 inhibits OSCC cell proliferation, invasion, migration, and stemness, and can partially reverse the tumor-promoting effect of lipopolysaccharide (LPS). This mechanism involves the inhibition of AKT activation and EMT (79). Furthermore, CX3CL1 induces innate and adaptive immunity, including the recruitment of effector cells of CD57+ lymphocytes to tumor tissues (80). This functional complexity has significant implications for developing CX3CL1-targeted therapeutic strategies in OSCC. Consequently, designing drugs that selectively enhance CX3CL1’s antitumor functions while inhibiting its pro-oncogenic activities presents a considerable challenge.

## 3 Mechanisms of chemokine regulation of the OSCC TME

The TME plays a critical role in tumorigenesis by harboring tumor cells that dynamically interact with surrounding components via the circulatory and lymphatic systems. These constant interactions decisively influence cancer initiation, progression, metastasis, and therapeutic response (124). In the TME, chemokines are crucial modulators, shaping key processes such as immune evasion, angiogenesis, lymphatic metastasis, and metabolic reprogramming.

### 3.1 Immune escape

**Figure 1:**
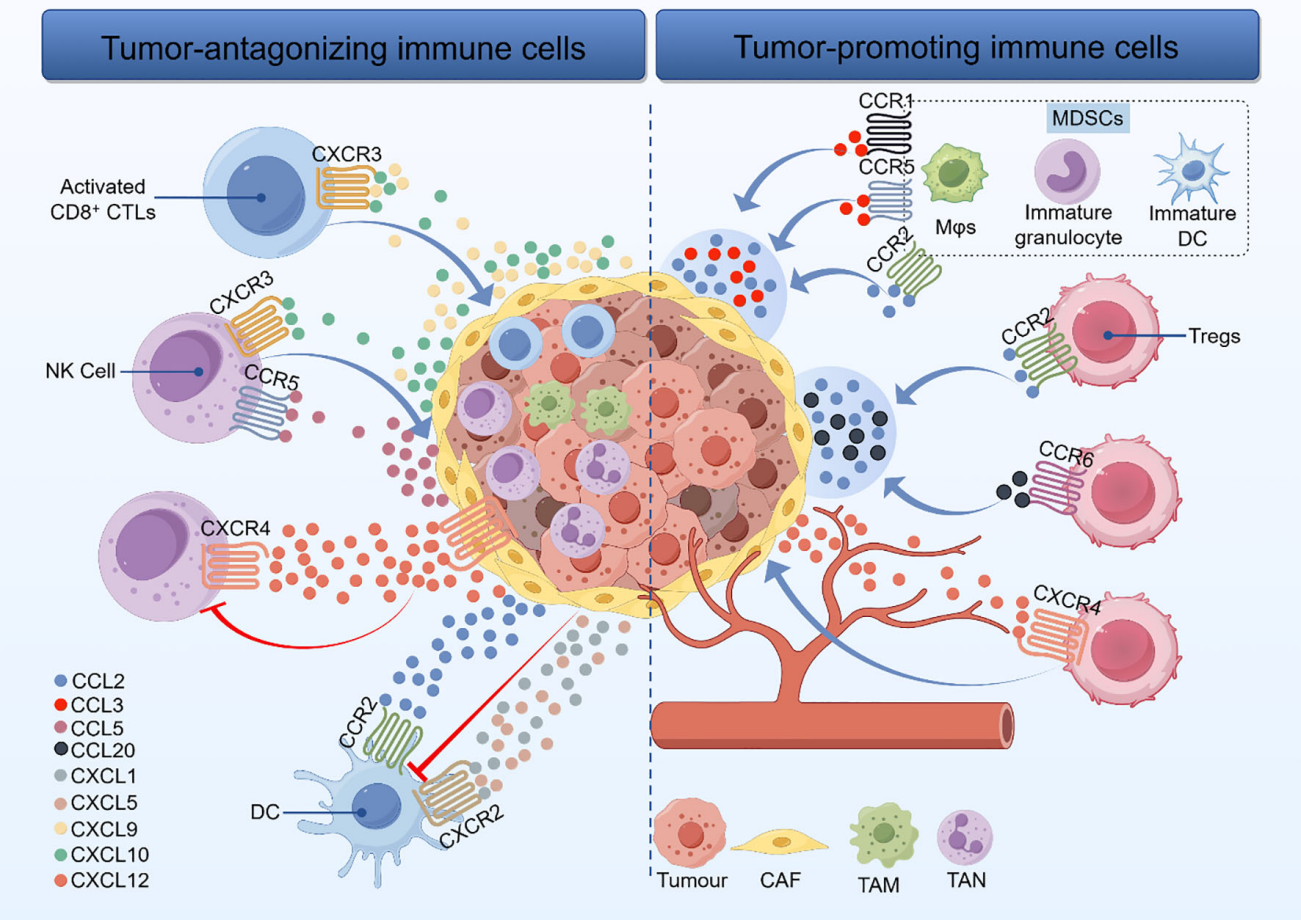
Relationship between tumor-antagonistic versus tumor-promoting immune cells and their chemokine and receptor networks in the immune microenvironment of oral squamous cell carcinoma. CAF: Cancer-Associated Fibroblasts; TAM: Tumor-Associated Macrophages; TAN: Tumor-Associated Neutrophils; NK Cell: Natural Killer cell; CD8^+^CTLs: CD8-positive Cytotoxic T Lymphocytes; Tregs: Regulatory T cells; DC: Dendritic Cells.

Tumor-associated immune cells exhibit dual functionality, capable of either antagonizing or promoting tumor progression(**Figure 1**). During early tumorigenesis, antitumor immune cells within the TME eliminate malignant cells through cytotoxic activity. However, as the disease progresses, neoplastic cells evade immune surveillance by suppressing effector immune functions via immunosuppressive mechanisms. Immune escape is thus recognized as a hallmark of cancer development (125). In this review, we examine the biological functions of immune cells within the TME, their roles in cancer immunotherapy, and their interactions with chemokines.

#### 3.1.1 Tumor-antagonizing immune cells

Effector T cells primarily comprise CD8+ CTLs and effector CD4+ T cells. CTLs, which recognize cancer cells presenting major histocompatibility complex class I (MHC-I) molecules, have long been considered the principal lymphocyte subset responsible for tumor cell killing (126). Guided by the chemokines CXCL9 and CXCL10, activated CTLs can migrate into the TME by expressing CXCR3 and exert cytotoxic antitumor effects (127). Studies indicate that CD8+ T cell infiltration is more prevalent in well-differentiated OSCC specimens and negatively correlates with disease progression (128). This suggesting that chemokine-mediated recruitment of CD8+ T cells may inhibit OSCC deterioration. This recruitment is a prerequisite for CD8+ T cell-mediated regulation of the TME. Furthermore, modulating inhibitory immune checkpoints represents another strategy to reduce cancer cell immune escape. In some tumors, cancer cells evade CTL-mediated killing by expressing ligands that engage inhibitory checkpoints on T cells, a key immune escape mechanism. Common inhibitory checkpoints implicated in OSCC include CTLA-4, PD-1, TIM-3, LAG-3, TIGIT, and CD96 (129). Consequently, developing drugs targeting PD-1, CTLA-4, and other inhibitory checkpoints is a promising therapeutic approach for OSCC. As summarized above, while chemokines can recruit CTLs to improve the TME, there is a lack of research to argue whether chemokines can influence inhibitory checkpoints to improve the immune escape of tumor cells, which is worthy of further experimental study and exploration.

NK cells constitute a critical antitumor immune subpopulation, providing chemokine-mediated immune surveillance against tumors. Chemokines, particularly CCL5, guide NK cell recruitment to inflammatory or cancerous sites to enhance antitumor activity (130). In OSCC, CCL5 is secreted by tumor or stromal cells – typically exhibits downregulation. It attracts NK cells via their CCR5 receptor. Similarly, CXCL10 recruits NK cells to tumors by binding CXCR3 on their surface, and its high expression correlates positively with favorable patient prognosis. Beyond recruitment, chemokines modulate NK cell activity by influencing tumor cell proliferation (131). For instance, CXCL12 binding to CXCR4 promotes tumor cell proliferation while suppressing NK cell infiltration. Notably, CXCL12 antagonists can restore NK cell function. In the TME, NK cells share functional parallels with CD8+ T cells. Both cell types are regulated by inhibitory immune checkpoints (e.g., CTLA-4, PD-1, Tim-3, KIR2DL-1/2/3, NKG2A, CD96, TIGIT), whose signaling restricts their cytotoxic potential. Consequently, blocking these inhibitory pathways represents a promising strategy to restore the antitumor function of NK cells for OSCC treatment.

DCs are specialized antigen-presenting cells (APCs) that present antigens and deliver stimulatory signals essential for T cell activation. Infiltration of mature, activated DCs into tumors enhances immune activation and effector cell recruitment. However, tumors can suppress DC function. For example, tumor-derived conditioned medium containing high levels of CCL2, CXCL1, and CXCL5 inhibits DC maturation *in vitro (*132). This suggests that within the TME, elevated concentrations of these chemokines contribute to immune escape by impairing DC antigen presentation. As mentioned above, mature DCs play a vital role in modulating the oral TME. Maintaining their function is closely linked to chemokine signaling. Additionally, inhibitory immune checkpoints critically regulate DC activity. Tumors can paralyze DCs through the induction of PD-1 expression. It is important to understand the mechanisms underlying regulation of PD-L1 expression in the OSCC microenvironment (133). PD-L1 expression is lower in high-grade invasive OSCC cell lines compared to low-grade invasive lines. Consequently, DC-based vaccines loaded with patient-specific neoantigens either alone or combined with inhibitory immune checkpoint blockade to restore DC antigen-presenting function represent promising immunotherapeutic strategies for OSCC.

#### 3.1.2 Tumor-promoting immune cells

The multifaceted role of Tregs makes them a critical factor in the progression and immune evasion mechanisms of OSCC (134). Chemokines are essential in orchestrating the immune response to cancer by guiding Treg cells to the TME. Key chemokine receptors, such as CCR2, CCR6, CCL20 and CXCR4, have been identified as pivotal contributors to Treg recruitment at tumor sites (135). These pathways enable Treg migration into the TME in response to CC and CXC chemokines, thereby shaping the intratumoral immune landscape. The distinct receptor profiles expressed by Tregs facilitate their rapid mobilization to inflammatory sites, underpinning their potent immunoregulatory function. Furthermore, the dynamic expression of these receptors, particularly within secondary lymphoid tissues, underscores their importance in Treg trafficking and activity (136). Elevated CCR6 expression in tumor-associated Tregs correlates with OSCC progression. CCL20, a ligand for CCR6, draws CCR6-expressing Tregs to the TME, driving tumor growth and worsening patient prognosis. OSCC screening based on salivary CCL20 expression enabled successful differentiation between patients with OSCC (49). Therefore, CCL20 expression may be a useful biomarker for OSCC. CXCR4 also play significant roles in mobilizing Tregs to tumor locations in OSCC (104). In conclusion, chemokine-receptor interactions critically govern Treg migration to tumors. Modulating this recruitment axis represents a potential strategy to enhance immunotherapy efficacy and halt disease progression in OSCC.

Myeloid-derived suppressor cells (MDSCs) represent a heterogeneous population of immature myeloid cells, comprising two primary subtypes: polymorphonuclear MDSCs (PMN-MDSCs) and monocytic MDSCs (M-MDSCs), which exhibit distinct phenotypic markers and morphological features (137). In recent years, MDSCs have garnered substantial attention due to their critical role as major inhibitors of anti-tumor immune responses and as key factors limiting the efficacy of cancer immunotherapy. Derived from myeloid progenitor cells, MDSCs encompass immature macrophages (Mφs), immature granulocytes, and immature DCs. CCL2、CCL3、CCL15、CXCL1 are predominantly secreted by various cell types, including Mφs, DCs, astrocytes, microglia, endothelial cells, keratinocytes, mesangial cells, fibroblasts, osteoblasts, and neurons. Notably, tumors such as OSCC, prostate cancer and breast cancer also produce CCL2. This indicates that MDSCs induce immune escape by producing CCL2、CCL3、CCL15、CXCL1.CXCL8, also known as IL-8, is produced by Mφs, endothelial cells, epithelial cells. Through its receptors CXCR1 and CXCR2, CXCL8 induces the recruitment of neutrophils, Mφs, DCs, mast cells, endothelial cells, and keratinocytes. CCL21 mediates the recruitment of B cells, T cells, DCs, and microglia via CCR7. CXCL13, also known as B lymphocyte chemoattractant (BLC) or B cell-attracting chemokine 1 (BCA-1), is produced by DCs, T follicular helper cells, prostate cancer cells, Jurkat cells, and cancer-associated myofibroblasts. It primarily attracts B cells and skin-derived migratory DCs through its receptor CXCR5. In summary, tumor-derived chemokines like CCL2 and CCL3 recruit MDSC precursors, macrophages, and DCs in OSCC. This promotes MDSC formation and ultimately suppresses antitumor immune responses.

### 3.2 Blood vessel formation and maturation

**Figure 2:**
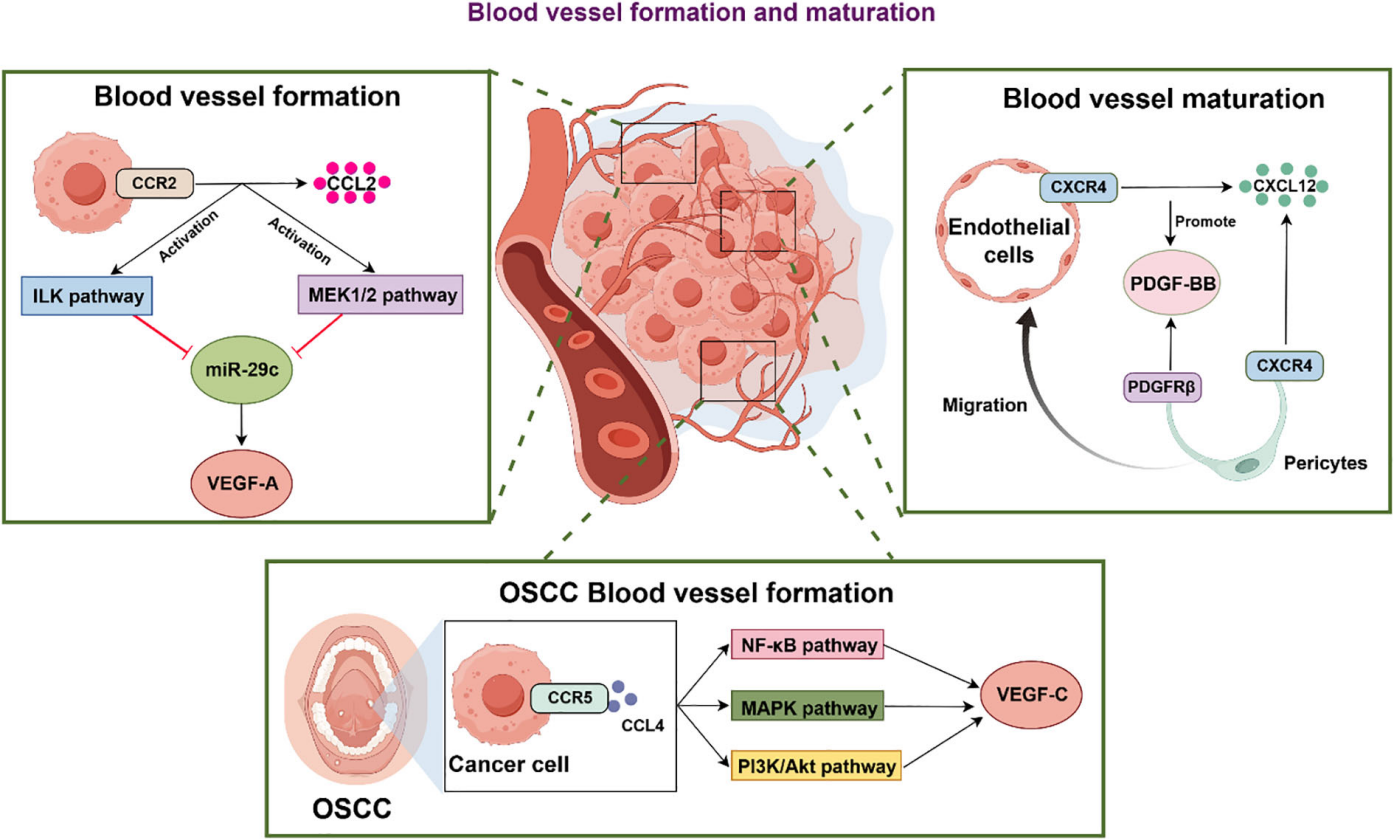
Chemokines and their receptors play important regulatory roles in angiogenesis and maturation of OSCC. OSCC: Oral Squamous Cell Carcinoma; ILK pathway: Integrin-linked kinase pathway; MEK1/2 pathway: Mitogen-activated protein kinase kinase 1/2 pathway; NF-κB pathway: Nuclear Factor kappa-light-chain-enhancer of activated B cells; miR-29c: microRNA-29c; MAPK pathway: Mitogen-Activated Protein Kinase pathway; PI3k-Akt pathway: Phosphatidylinositol 3-kinase - Protein kinase B pathway; VEGF-A: Vascular Endothelial Growth Factor-A; VEGF-C: Vascular Endothelial Growth Factor-C; PDGF-BB: Platelet-Derived Growth Factor-BB; PDGFRβ: Platelet-Derived Growth Factor Receptor β.

Maintaining the TME requires neovascularization, driven primarily by upregulation of pro-angiogenic factors that ensure adequate oxygen and nutrient supply (**Figure 2**).

(In the process of vascularization, CCL2 binding to CCR2 on tumor cells triggers ILK and MEK1/2 pathways, inhibits miR-29c to promote the generation of VEGF-A, and further promotes the formation of neovascularization. On the other hand, CCL4 activates CCR5 of cancer cells to up-regulate the expression of VEGF-C through signaling pathways such as NF-κB, MAPK and PI3K/Akt, driving tumor vascularization. In the process of vascular maturation, the up-regulation of PDGF-BB expression is the core link to promote the stability and functional improvement of vascular structure. Specifically, CXCR4 on the surface of endothelial cells and pericytes can bind to the chemokine CXCL12, and this interaction will promote the expression of PDGF-BB; subsequently, PDGFRβ on the surface of pericytes specifically binds to PDGF-BB, driving pericytes to migrate to endothelial cells and bind to them, thereby forming a stable vascular wall structure, and finally pushing blood vessels to complete the maturation process).

Chemokines critically regulate this process by mediating expression of angiogenesis-related factors (138). VEGF is closely related to the proliferation of vascular endothelial cells and is a key factor in the promotion of neoangiogenesis by chemokines (139). CCL2 activates ILK and MEK1/2 signaling pathways and downregulates miR-29c to promote VEGF-A expression in OSCC by specifically binding to CCR2 receptors on the cell surface, and is a new molecular therapeutic target to inhibit neovascularization and metastasis in OSCC (42). Similarly, Elevated serum levels of CCL4 correlate with the progression of advanced OSCC. Mechanistically, CCL4 binds to CCR5 receptors on oral cancer cells, activating the NF-κB, MAPK, and PI3K/Akt signaling pathways. This molecular interaction induces VEGF-C expression both *in vitro* and *in vivo*. Collectively, these findings suggest that CCL4 holds potential as a molecular target for OSCC intervention (140). Beyond angiogenesis, chemokines facilitate vascular maturation. CXCL12 can not only specifically bind to CXCR4 and directly recruit endothelial progenitor cells to the site of angiogenesis, stimulate the proliferation and migration of pericytes, but also stimulate the binding of PDGF-BB and PDGF-Rβ to indirectly promote the arrangement of pericytes and vascular smooth muscle cells around the blood vessel wall, enhancing the integrity and mechanical strength of vascular structure (26). This will maintain the stability of pericytes and ensure the blood supply to the tumor. Consequently, chemokines coordinately regulate both vessel formation and maturation in OSCC. Therapeutic strategies targeting these pathways should suppress peritumoral vasculature development.

### 3.3 Matrix degradation and fibrosis

#### 3.3.1 Degradation of the extracellular matrix

**Figure 3:**
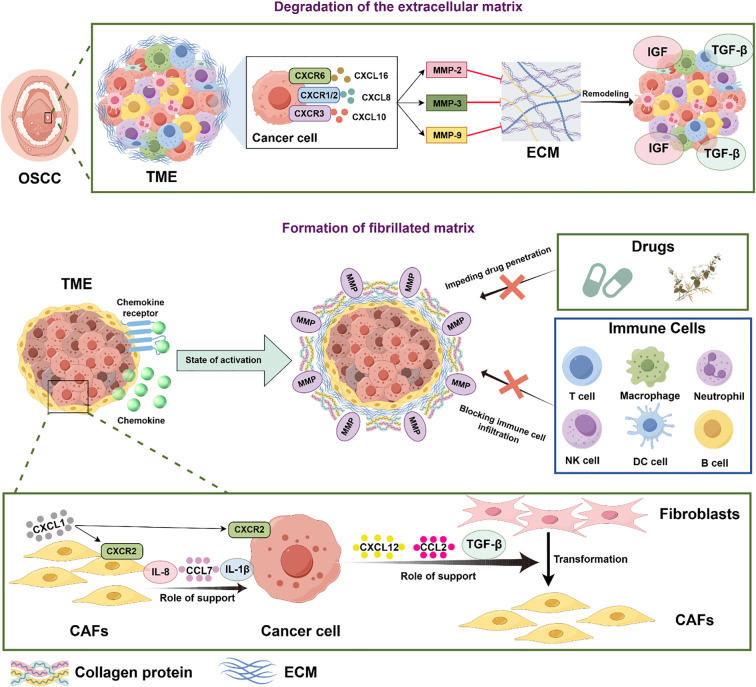
Mechanisms of chemokines and their receptors in extracellular matrix degradation and fibrotic matrix formation in oral cancer. OSCC: Oral Squamous Cell Carcinoma; TME: Tumor Microenvironment; MMP-2: Matrix Metalloproteinase-2; MMP-3: Matrix Metalloproteinase-3; MMP-9: Matrix Metalloproteinase-9; ECM: Extracellular Matrix; IGF: Insulin-like Growth Factor; TGF-β: Transforming Growth Factor-β; CAFs: Cancer-Associated Fibroblasts.

The formation of the TME is closely linked to the extracellular matrix (ECM). Degradation of the ECM enables tumors to break through the basement membrane and surrounding matrix, facilitating invasion into adjacent tissues and metastasis to distant sites (**Figure 3**).

(On the one hand, OSCC cells activate the expression and release of matrix metalloproteinases MMP-2, MMP-3, MMP-9 by binding chemokines such as CXCL8, CXCL10, and CXCL16 to receptors CXCR1/2, CXCR3, and CXCR6, respectively, breaking the dynamic balance of the extracellular matrix and degrading ECM to open up a path for tumor invasion and metastasis. On the contrary, some chemokines bind to their receptors and actively promote the formation of a fibrotic matrix. This fibrotic matrix not only constitutes a physical barrier, hindering the penetration of cancer therapeutic drugs into tumor tissues and weakening the therapeutic effect; it also blocks the infiltration of immune cells (such as T cells, NK cells, etc.) into the tumor microenvironment and inhibits the killing effect of the immune system on tumor cells, thereby helping tumors escape treatment and immune attack. Fibroblasts of cancer cells play an important role in this process.CXCR2 receptors on the surface of CAFs can bind to self-secreted CXCL1, and can also release IL-8, CCL7, IL-1β, etc., providing support for cancer cells; at the same time, CXCL12, CCL2, and TGF-β secreted by cancer cells play a supporting role, promoting the transformation of normal fibroblasts into CAFs.

Key enzymes mediating ECM degradation are the MMPs. These proteolytic enzymes, secreted by both normal and cancer cells, consist of a prodomain, a catalytic region, a hinge region, and a hemopexin domain (141). Chemokines contribute to OSCC development by promoting MMP expression. For instance, mRNA expression of CXCR1 and CXCR2 is observed in both normal oral keratinocytes (NOKs) and oral cancer cell lines (OCCLs), with significantly higher protein levels in OCCLs. CXCR2 stimulation increases MMP-9 release from both cell types, with OCCLs releasing significantly higher amounts (142). CXCL8 and CXCL10 promote ECM degradation by inducing overexpression of MMP-2 and MMP-9, thereby creating a gateway for tumor cell invasion (143). Similarly, CXCR6 drives OSCC cell proliferation by inducing MMP3 secretion, actively remodeling the ECM (144). Metalloproteinases activities are tightly regulated by proteolytic activation and inhibition via their natural inhibitors, tissue inhibitors of metalloproteinases (TIMPs), and the imbalance of the activation and inhibition is responsible in progression or inhibition of OSCC (145). However, the relationship between chemokines and TIMPs is less explored in the literature. Therefore, future research should investigate whether chemokines enhance MMP activity by modulating TIMP expression, thereby promoting ECM degradation and exacerbating OSCC.

#### 3.3.2 Formation of fibrillated matrix

The dense fibrotic stroma barrier impedes drug penetration and immune cell infiltration, thereby regulating the TME. CAFs, the most common cell type involved in fibrotic stroma formation, are fibroblasts co-opted to support cancer cells (146). Chemokines can activate CAFs, prompting them to secrete collagen, fibronectin, and MMPs to promote fibrotic stroma barrier formation and modulate the TME. Notably, CXCL1 and its receptor CXCR2 are highly expressed in OSCC. This CXCL1/CXCR2 axis in CAFs mediates cancer cell invasion via induction of IL-1β, revealing a reciprocal dependency between CAFs and cancer cells within the OSCC microenvironment (61). Activated CAFs exhibit increased expression of pro-inflammatory genes, including CXCL1, CXCL2, IL-1β, and IL-6. CXCL1 can further elevate CAF levels via an autocrine mechanism (62). Studies confirm that cytokines secreted by CAFs, such as CCL7, CXCL1, and IL-8 (CXCL8), promote human OSCC development (147). Experimentally, inhibiting CAF-conditioned medium reduced OSCC differentiation levels compared to controls, an effect likely mediated by decreased CCL2 production. In summary, chemokines promote OSCC progression by activating CAFs, and CAFs, in turn, promote OSCC by secreting chemokines.

## 4 Potential chemokine and chemokine receptor therapies that can be used to treat OSCC

One study demonstrated that 3,3’,4’,5’-Tetramethoxychalcone (TMC) inhibits the proliferation and migration of human oral cancer cells via p53-mediated mitochondria-dependent apoptosis. In this process, TMC attenuates the CCL5/CCR5 axis, thereby reducing the migration potential of OSCC cells (148).

SB225002 is an antagonist of the chemokine receptor CXCR2, mainly blocking the signaling pathways of chemokines (such as CXCL8, CXCL1, CXCL2, etc.) that act through this receptor. Overactivation of the epidermal growth factor receptor (EGFR) pathway and chronic inflammation are common features of OSCC. Studies have shown that CXCL1-CXCR2 axis-targeted drugs SB225002 more effective in overcoming EGFR activation than IL-1β-targeted regimens (149). Takenobu et al. reported that IL-1β triggered the detachment of the extracellular domain of pro-HB-EGF in vero cells. This mechanism may occur in OSCC cells and explains why CXCR2 inhibitors SB225002 not completely inhibit IL-1β-mediated EGFR tyrosine phosphorylation (150). These studies indicate that a combination of EGFR TKIs with a regimen targeting the CXCL1-CXCR2 axis may be a promising therapeutic strategy for OSCC patients with over-expressed EGFR.

In a study on CXCL1 promoting the mutual activation of cancer-associated fibroblasts and OSCC cells, the results showed that CXCL1 neutralizing antibody and CXCR2 antagonist SB225002 attenuated the invasion-promoting effect of CAF-CM, confirming the importance of CXCL1 in tumor invasion (61). Another study showed that the application of SB225002 and cisplatin could inhibit CD10H and CD10L HN6 tumor cells. CD10H HN6 is more sensitive to SB225002, and its resistance to cisplatin is higher than that of CD10L HN6. It is hypothesized that targeting IL8 (associated with tumor cell migration, invasion, and chemoresistance) may increase the effectiveness of cisplatin in the treatment of OSCC. In this process, SB225002 play a role in suppressing tumor cells (151).

It is worth noting, CXCR4 plays a key role in tumor angiogenesis required for OSCC progression. AMD3100 is a specific antagonist that blocks the binding pocket of CXCR4. TAITN is a novel and unique subtype of necrosis. Due to significant inhibition of the tumor vasculature, island tumor masses limited by the inhibited blood vessels remain in the necrotic tissue. CXCR4 antagonist-induced TAITN may be an effective anti-angiogenic therapy strategy for the treatment of OSCC. The data from this study could open up CXCR4-targeted personalized therapies for CXCR4-positive tumor vessels (152). Studies on the CXCL12-CXCR4/CXCR7 axis and OSCC have shown that AMD3100 can also affect the migration and invasion ability of oral cancer cells (153).

Furthermore, results from an additional *in vivo* and *in vitro* study indicated that monotherapy with cisplatin yields limited tumor suppression in oral squamous cell carcinoma. In contrast, the combination of cisplatin and AMD3100 exhibited potential therapeutic benefits, such as reduction in tumor volume, decreased morbidity at the resection site, and enhanced patient quality of life. This combinatorial regimen may thus represent a promising strategy for managing refractory OSCC. The anti-tumor properties of AMD3100 observed in this study underscore its compatibility with existing anticancer agents and molecularly targeted therapies. Moreover, its ability to counteract resistance to VEGF inhibitors highlights the clinical potential of this drug combination, offering new therapeutic avenues for OSCC patients (154).

In TCA8113 and SCC-9 cells treated with CXCR4, TN14003, and CXCR4-LV1-specific inhibitors, the number of cells in the G1 phase was significantly increased, while the number of cells in the S phase and G2/M phases was significantly decreased. This suggests that downregulation of CXCR4 can induce G1 phase arrest and apoptosis. and inhibit the proliferation of tumor cells (155).

The CCR5/CCL5 axis is essential for the interaction between malignant cells and components of the microenvironment. However, the role of CCR5 antagonists in OSCC has not been well studied. The results showed that the expression of CCR5 was associated with clinical stage and metastasis, while the expression of CCL5 was associated with overall survival. shRNA and Maraviroc can reduce cell proliferation in a dose-dependent manner. Maraviroc treatment also increased apoptosis and improved cytoskeletal organization (48). A separate study showed that zoledronic acid (ZA) effectively inhibited the malignant phenotype of OSCC cells in an organotypic raft culture model, manifested by anchor-independent growth and a significant attenuation of epithelial hyperplasia. The compound also inhibits cancer stem cell (CSC)-related properties, in particular reducing self-renewal ability, migration potential, and chemoradiotherapy resistance. Mechanistic studies have shown that ZA exposure significantly downregulates the expression of CCL3 in OSCC cells. Subsequent pharmacological intervention with CCL3 receptor antagonists effectively reversed CCL3-mediated maintenance of CSC properties. Notably, exogenous CCL3 supplementation restored the CSC phenotype in ZA-treated cells, confirming the critical role of the CCL3 signaling axis in mediating the therapeutic effects of ZA on CSC regulation (156).

Currently, the development of chemokine antagonists is a promising cancer treatment. However, chemokine antagonists that can be used to intervene in the malignant progression of OSCC are relatively few, and further exploration is still needed (**Table 2**).

**Table 2:** Treatment methods targeting chemokines and chemokine receptors in oral squamous cell carcinoma.

Target	Treatment methods	Result	Ref.
CCL5	3,3’,4’,5’-Tetramethoxychalcone (TMC)	TMC inhibited OSCC proliferation (IC50 = 1.0-4.5 μM) by inducing G2/M arrest and DNA damage (γ-H2AX↑, Chk2↑). It triggered mitochondrial apoptosis via p53/BAK/BAX activation and BCL-2 suppression. TMC also attenuated migration by disrupting the CCL5/CCR5 axis.	(148)
CXCR2	SB225002	As a CXCR2-specific antagonist, SB225002 inhibits IL-1β -mediated EGFR trans activation by blocking the CXCL1-CXCR2 axis, thereby significantly reducing tyrosine phosphorylation of EGFR in OSCC cells (within 20 minutes).	(149)
		SB225002 significantly inhibits the migration of SAS cells induced by CXCL1. Similarly, the conditioned medium from CAFs also increased cell migration, and this effect was weakened in a dose-dependent manner after treatment with SB225002. MMP-1 may be a downstream effector of invasion in the OSCC microenvironment mediated by CXCL1/CXCR2.	(61)
CXCR4	AMD3100	A CXCR4 Antagonist AMD3100 Induced Tumor Necrosis in OSCC-Xenotransplanted Mice. CXCR4 antagonist triggered TAITN in OSCC tumors.	(152)
		The CXCR4 antagonist AMD3100 dose-dependently inhibited OSCC proliferation (10 ng/mL-1 μg/mL) and significantly suppressed CXCL12-induced migration/invasion (p<0.05). Combined AMD3100/CXCR7 knockdown showed no additive effect, indicating functional redundancy in CXCR4/CXCR7 signaling. These findings position AMD3100 as a promising anti-metastatic agent for CXCR4+ OSCC, warranting further *in vivo* validation of this therapeutic strategy.	(153)
CXCR4	AMD3100 + Cisplatin	The findings of this *in-vivo* and *in-vitro* study suggest the potential efficacy of combining AMD3100 with cisplatin for treating OSCC in cases where the tumor-suppressive effect of cisplatin alone is limited. The amalgamation of cisplatin and a CXCR4 inhibitor may provide a promising novel approach for managing refractory OSCC.	(154)
CXCR4	TN14003	In this study, cells of phase G_1_ significantly increased, and cells of S and G_2_/M phases significantly decreased in cells treated with TN14003 and CXCR4-LV1, suggesting that CXCR4 downregulation could induce phase G_1_ arrest and apoptosis as well as inhibit tumor cell proliferation.	(155)
CCR5	Maraviroc	CCR5 expression correlated with tumor stage/metastasis, while CCL5 linked to survival. Exogenous CCL5 boosted proliferation, whereas shRNA and Maraviroc suppressed it dose-dependently. Maraviroc also enhanced apoptosis, altered cytoskeleton, and reduced neck Metastasis *in vivo*.	(48)
CCL3	Zoledronic Acid (ZA)	ZA exhibited antitumor effects in OSCC by inhibiting malignant growth and suppressing CSC properties, including self-renewal, migration, and chemo-radioresistance. Notably, ZA downregulated CCL3, and CCL3 supplementation reversed ZA’s effects. These findings suggest ZA as a potential therapy for oral cancer by targeting cancer stemness.	(156)

We have also supplemented clinical trial results for chemokine-targeted drugs across multiple cancers to better explore their potential value in treating oral squamous cell carcinoma. As shown in **Table 3**:

**Table 3:** Clinical trial results of common chemokine-targeted drugs across multiple cancers.

Target	Treatment	Tumor	Name (Recruitment Status)	ClinicalTrials.gov identifier
CCR5	Maraviroc	Colorectal cancer	Treatment of Advanced Colorectal Cancer Patients With Hepatic Liver Metastases Using the CCR5-Antagonist Maraviroc (Phase I Maracon Trial).	NCT01736813
	Maraviroc + pembrolizumab	Colorectal cancer	A Phase I Trial of Combined Pembrolizumab and Maraviroc for the Treatment of Refractory Microsatellite Stable (MSS) Metastatic Colorectal Cancer.	NCT03274804
	Maraviroc	Kaposi’s sarcoma	Effects of Maraviroc on HIV-related Kaposi’s Sarcoma.	NCT01276236
CXCR4	Plerixafor(AMD3100) + cemiplimab,	metastatic pancreatic adenocarcinoma	A phase 2 trial of plerixafor and cemiplimab in metastatic pancreatic adenocarcinoma reveals recruitment of T cells but also immunosuppressive macrophages.	NCT04177810
	Plerixafor + G-CSF	Non-Hodgkin Lymphoma or Multiple Myeloma	A Phase II Study of Plerixafor, Combined with G-CSF for CD34(+) Cell Mobilization in Patients with Non-Hodgkin Lymphoma or Multiple Myeloma Undergoing Autologous Transplantation.	
	Plerixafor + Bortezomib	Relapsed/Refractory Multiple Myeloma	A Phase I/II Trial of the Plerixafor Combined with Bortezomib to Overcome Therapeutic Resistance in Relapsed/Refractory Multiple Myeloma.	NCT00903968
	Plerixafor + G-CSF	Non-Hodgkin’s Lymphoma	A Phase III, Multicenter, Randomized, Placebo-Controlled Trial of Plerixafor, Plus G-CSF for Hematopoietic Stem Cell Mobilization in Non-Hodgkin’s Lymphoma Patients Undergoing Autologous Transplantation.	
	Plerixafor	multiple myeloma	A Phase II Study of Single-Agent Plerixafor, a CXCR4 Antagonist, for Hematopoietic Stem Cell Mobilization in Patients with Multiple Myeloma.	NCT00396383
CCR4	Mogamulizumab + Anti-PD-1 Nivolumab	Operable Solid Tumors	A Phase I Trial of Preoperative Combination Therapy with the Anti-CCR4 Antibody Mogamulizumab and Anti-PD-1 Nivolumab in Patients with Operable Solid Tumors.	NCT02946671
	Mogamulizumab + KHK2455	Advanced Solid Tumors	A Phase I Trial of the IDO1 Inhibitor KHK2455 in Combination with the Anti-CCR4 Monoclonal Antibody Mogamulizumab for Patients with Advanced Solid Tumors.	NCT02867007
	Mogamulizumab	Relapsed or Refractory Cutaneous T-Cell Lymphoma	A Phase 1/2 Study of the Anti-CCR4 Monoclonal Antibody Mogamulizumab in Patients with Relapsed or Refractory Cutaneous T-Cell Lymphoma.	NCT00888927
	Mogamulizumab	Relapsed Peripheral T-Cell Lymphoma and Cutaneous T-Cell Lymphoma	A Phase II Study of the Anti-CCR4 Antibody Mogamulizumab in Patients with Relapsed Peripheral T-Cell Lymphoma and Cutaneous T-Cell Lymphoma.	NCT01192984
CCR2	PF-04136309 + FOLFIRINOX Chemotherapy	Borderline Resectable or Locally Advanced Pancreatic Ductal Adenocarcinoma	A Phase Ib Study of the CCR2 Inhibitor PF-04136309 in Combination with FOLFIRINOX Chemotherapy for Patients with Borderline Resectable or Locally Advanced Pancreatic Ductal Adenocarcinoma.	NCT01413022
	PF-04136309 + Nab-Paclitaxel + Gemcitabine	Metastatic Pancreatic Ductal Adenocarcinoma	A Phase Ib Study of the CCR2 Inhibitor PF-04136309 in Combination with Nab-Paclitaxel and Gemcitabine for Patients with Metastatic Pancreatic Ductal Adenocarcinoma.	NCT02732938
	Carlumab (CNTO 888)	Castration-Resistant Prostate Cancer	A Phase II Study of the Anti-CCL2 Monoclonal Antibody Carlumab in Patients with Metastatic Castration-Resistant Prostate Cancer.	
CXCR1	Reparixin	HER-2-Negative Operable Breast Cancer	A Phase II Study of the CXCR1 Inhibitor Reparixin as Preoperative Therapy for Patients with HER-2-Negative Operable Breast Cancer.	NCT01861054
	Reparixin	Metastatic Breast Cancer	A Phase Ib Study of the CXCR1/2 Inhibitor Reparixin in Combination with Paclitaxel for Metastatic Breast Cancer.	NCT02001974
CXCR2	Navarixin	Advanced Castration-Resistant Prostate Cancer, Microsatellite-Stable Colorectal Cancer, or Non-Small-Cell Lung Cancer	A Phase II Study of the CXCR2 Antagonist Navarixin in Combination with Pembrolizumab for Patients with Advanced Castration-Resistant Prostate Cancer, Microsatellite-Stable Colorectal Cancer, or Non-Small-Cell Lung Cancer.	NCT03473925

## 5 Future challenges and research directions

### 5.1 Spatio-temporal dynamics of chemokine networks

Now, extensive studies have shown that chemokines can be used as biomarkers, reflecting the malignant progression (157–161) and metastasis of cancer (157, 159, 162–164), or as indicators for differential diagnosis (164–166).

Research indicates that CXCL1 expression in bladder urothelial carcinoma (UCB) exhibits significant clinical heterogeneity. Specifically, CXCL1 is highly expressed in high-grade and high-stage tumor tissues. It is secreted to the tumor-stroma interface via paracrine pathways or leaks into the systemic circulation through tumor-associated blood vessels (165). Furthermore, CXCL1 signaling within the TME may contribute to repeated intravesical recurrence and disease progression. Results from another study on colorectal cancer (CRC) demonstrated that plasma CXCL3 levels are associated with tumor progression and poor prognosis in CRC patients. This suggests plasma CXCL3 may serve as a novel diagnostic and prognostic biomarker for CRC (161).

Notably, Platelet Factor 4 (PF4), also known as chemokine (C-X-C motif) ligand 4 (CXCL4), exhibits high expression on macrophages in endometriosis but low expression on TAMs in clear cell ovarian cancer (166). This differential expression suggests that CXCL4 levels could potentially aid in distinguishing benign endometriosis from early malignant transformation. The research by Monika et al. conducted statistical analysis using the clinical data of patients and the obtained indicators, and determined that some of these indicators had a diagnostic utility much higher than that of the currently most commonly used tumor markers (CEA). Moreover, two of these indicators (CXCL14/CEA and CXCL16/CEA) not only show extremely high usefulness in the early detection of CRC, but also can determine whether the stage is low (stage I and II) or high (stage III and IV) (160).

The latest research indicates that CCLs present a dynamic regulatory network and dual roles in CRC. Studies have found that the expressions of CCL3, CCL4, and CCL26 in CRC tissues are significantly upregulated, while CCL2, CCL5, CCL11, CCL21, and CCL28 are downregulated. Among them, the high expression of CCL4/CCL11/CCL28 is closely related to the prolonged survival period of patients, suggesting its potential for tumor suppression. Low expression of CCL5/CCL21 is associated with advanced stage and poor prognosis. Mechanism, CCLs regulate tumor proliferation and the immune microenvironment through Wnt, Toll-like receptor signaling pathways and kinases (such as SRC family, CDK2). Among them, RELA/NFKB1 mediates transcriptional regulation and drives the interaction network between CCL and immune cells (B cells, CD8+ T cells, macrophages, etc.). These findings highlight the potential of CCLs as multifunctional biomarkers for CRC staging diagnosis, prognosis assessment, and targeted therapy (such as restoring CCL5/CCL21 expression or inhibiting the CCL3/CCR5 axis), providing a new direction for immune-kinase combination therapy (17).

TAMs selectively secrete CCL18, which actively promotes the formation of an immunosuppressive TME. Notably, serum CCL18 levels are significantly elevated in non-small cell lung cancer (NSCLC) patients and exhibit a negative correlation with both prognosis and overall survival (167–169), suggesting its key role as a tumor-promoting chemokine. A study on laryngeal squamous cell carcinoma has shown that chemokine receptors CCR6/CCR7 and their ligands CCL19/CCL20 synergically drive cervical lymph node metastasis of laryngeal squamous cell carcinoma through differential expression and Treg recruitment (163). Collectively, these studies confirm the broad application potential of chemokines in cancer staging, prognosis assessment, and differential diagnosis. As multifunctional biomarkers, their clinical utility continues to be extensively explored.

A recent systematic review, analyzing 50 studies, revealed the core role of chemokines in OSCC and its prodromal lesions (OPMDs) (170). The CXCR4/CXCL12 axis is significantly associated with lymph node metastasis and poor prognosis. Its high expression suggests enhanced tumor invasiveness (171). CCR7 can promote the polarization of M2 macrophages (106), and its expression is upregulated in the primary lesion and metastatic lymph nodes of OSCC. It can be used as a diagnostic and prognostic marker and is closely related to tumor stage, lymphatic metastasis density and decreased survival rate (172–174). CXCL10 and CCL22 have been emphasized due to their strong prognostic and metastatic correlations (70, 175–177). They can promote microenvironment remodeling and drive immune escape by recruiting immunosuppressive cells (such as Treg). Furthermore, CXCR2 and XCR1 are associated with the metastatic potential at the forefront of tumor invasion (178–180). Among them, CXCR2 may be involved in the invasion and metastasis of OSCC by regulating actin cytoskeletal remodeling (179).

However, given the vastness of the chemokine family, fully elucidating its specific mechanisms remains a monumental task. Chemokines can simultaneously bind to multiple receptors, and signal output may be influenced by protein modifications. Nevertheless, related research in this area remains scarce.

The CCR2 gene encodes two isoforms: CCR2A and CCR2B. Due to alternative splicing, CCR2A and CCR2B exhibit distinct amino acid sequences in their intracellular C-terminal tails. The C-terminal Helix 8 of CCR2A has fewer basic residues, impairing cell surface localization. In contrast, CCR2B’s C-terminus is rich in Ser/Thr residues, likely phosphorylated by GRKs to recruit β-arrestin and drive endocytosis (181). Studies have also demonstrated that the N-terminal sequence of each CXCR4 variant determines receptor expression and influences ligand recognition. Additionally, CXCR4 variants may mutually regulate or interact with each other during CXCL12-stimulated cellular responses (182). The research findings of Yamina A. Berchiche et al. demonstrate that CXCR3 splice variants activate distinct signaling pathways and exhibit non-redundant functions, suggesting a mechanism for tissue specific biased agonism (183).

Future research should prioritize investigating the dynamic changes in chemokine expression across OSCC stages, subtypes, and microenvironmental compartments. Additionally, elucidating the mechanisms underlying chemokine dysregulation in OSCC and its specific role in driving metastasis is crucial. This understanding will be instrumental in identifying novel chemokine-based therapeutic targets for OSCC. Targeted chemokines may also trigger the activation of compensatory pathways (such as the upregulation of CCR7 after inhibiting CXCR4), and multi-target inhibitors need to be developed. At the same time, focus should also be placed on the variants of chemokine receptors to explore their possible unique roles.

Moreover, as previously mentioned, most current chemokine antagonists target inhibition of individual factors while neglecting isoform specificity or cross-talk between downstream pathways, resulting in limited efficacy or drug resistance. Future research should prioritize investigations in this direction.

### 5.2 Practical challenges in clinical transformation

The incidence of OSCC continues to increase every year. Chemotherapy is effective for advanced tumors, but the side effect of normal tissue damage remains a major problem. Targeted nanoparticle delivery systems are expected to overcome this obstacle. Chemokines, as key regulatory factors of the TME, play an important role in cancer progression, immune escape and metastasis. However, clinical translation of chemokine-targeted approaches in OSCC faces multifaceted hurdles, necessitating careful optimization of both therapeutic efficacy and safety profiles.

Inhibitors targeting chemokines have long been widely used in clinical practice, and the therapeutic prospects of small molecule antagonists such as Plerixafor, BKT140, LY2510924, PF-06747143, ulocuplumab and NOX-A12 have been emphasized in the literature (184). The use of CXCR4 antagonists has been proven to disrupt the interaction between tumors and the matrix (185). The clinical application potential of targeted imaging and treatment of CXCR4 using β or positron emitters labeled antagonists is great, and the side effects are very small (186). The development of relevant nanocarriers or locally sustained-release systems (such as hydrogels) targeting chemokine antagonists or agonists may enhance the targeting of drugs and reduce their systemic toxicity. A study on ovarian cancer shows that compared with other groups, targeting AMD-NP-PTX exhibits excellent cellular drug uptake, controlled drug release, significant anti-cancer efficacy and biodistribution, good biocompatibility and safety (187). It can be used as a tumor-targeted drug carrier to deliver PTX into ovarian cancer cells. It can also serve as an inhibitor of the CXCL12/CXCR4 axis to provide synergistic anti-cancer effects. An injectable hydrogel containing METTL3 inhibitor can inhibit the methylation of N6-methyladenosine RNA in CRC, resulting in increased expression of CXCL9 and CXCL10, and promoting the migration of CAR-NK cells to eliminate CRC cells (188). Effectively prevent the recurrence of CRC after surgery in CRC mouse models. It is worth noting that CCL2 can inhibit the normalization of induced blood vessels, while anti-CCL2 therapy can induce blood vessel normalization and improve tumor perfusion, thereby enhancing tumor-targeted drug delivery and anti-cancer nanotherapy (189). Perhaps in the future, emphasis can be placed on developing synergistic therapies that inhibit CCL2 and other chemokine-based tumor-targeted drug carriers. In fact, some scholars have also provided some insights into the potential therapeutic benefits of using novel delivery systems targeting the CCL2/CCR2 axis to treat cancer and inflammation (39).

Research has now created MOFs coated with DPSC membrane containing CXCR2, a novel MOF@DPSCM nanoparticle. The DPSC membrane can modify MOF to achieve the specific targeting ability of OSCC *in vitro* and *in vivo*, and has good biocompatibility. And these novel MOF-DOX@DPSCM nanoparticles can specifically and effectively inhibit the growth of OSCC *in vivo (*115). However, in the field of OSCC, the research on chemokine nanocarriers or local sustained-release systems is still insufficient and deserves further study.

For example, liver-targeted pTrap LCP nanoparticles significantly reduce the expression of CXCL12 in the liver by locally delivering the CXCL12 trap gene, inhibit liver metastasis and avoid systemic toxicity (190). Similar strategies can be borrowed for the local treatment of OSCC, but the influence of the complex oral microenvironment on the stability of nanoparticles needs to be addressed. Although non-viral vectors (such as calcium phosphate nanoparticles) have high safety, their transfection efficiency is low (191). They can be combined with new materials (such as MXene nanosheets (192)) to enhance targeting.

Long-term inhibition of chemokines may weaken the recruitment of immune cells such as CD8+ T cells and NK cells. For example, although targeting the CCL2/CCR2 axis can reduce the infiltration of TAMs (193), it may inhibit the protective effect of macrophages in infection or tissue repair. Patients with OSCC are prone to infection after surgery, and it is necessary to balance immunosuppression and anti-tumor effects. IL-2 therapy leads to immunosuppression by activating Treg cells, while the improved pegylated IL-2 (such as bempeg) selectively activates effector T cells, providing ideas for the combined immunotherapy of OSCC (194–196).

Future therapeutic development for OSCC should prioritize chemokine-targeted local therapies. Given the superficial location of OSCC lesions, localized delivery systems—such as oral patches containing CXCR4 inhibitors or injectable hydrogels—offer a promising strategy to minimize systemic exposure while enhancing treatment efficacy. Combining these approaches with immunotherapy may yield synergistic benefits. Notably, high CCR7 expression strongly correlates with lymph node metastasis in OSCC (54). Anti-ccr7 antibody combined with radiotherapy can inhibit metastasis and activate CD8+ T cells.

## 6 Conclusions and prospects

OSCC is characterized by insidious onset, high mortality, and poor prognosis, underscoring an urgent need for novel therapeutic strategies. In recent years, immunotherapy and molecular targeted therapy have gained significant traction. These approaches aim to remodel the TME and counteract immune tolerance. Chemokines represent promising molecular targets due to their critical roles in regulating immune cell trafficking, tumor-stroma interactions, and vascular remodeling within the TME.

In this review outlines the functions of chemokines and their receptors in OSCC immune responses, cancer progression, chemotherapy resistance, and their diagnostic/prognostic potential. We specifically elucidate the complex, often dualistic roles of key chemokines – including CCL2, CCL5, CCL20, CCL19/CCL21, CXCL1, CXCL8, CXCL12, and CX3CL1 series in OSCC pathogenesis, emphasizing their regulation of the TME. Additionally, we summarize potential chemokine/chemokine receptor-targeted therapies for OSCC.

Chemokines form intricate signaling networks with other tumor-derived factors. Combining chemokine antagonists with established anti-tumor therapies represents a promising strategy to prolong OSCC patient survival. Despite decades of research, the chemokine family’s large size means mechanisms in OSCC remain incompletely understood. Current research exhibits a pronounced imbalance: CXC chemokines (α chemokines) and CC chemokines (β chemokines) subfamilies are relatively well-studied, while others remain largely unexplored. Furthermore, evidence supporting the clinical application of chemokine-targeted diagnostics or therapeutics in OSCC remains limited. Consequently, this review cannot extensively elaborate on clinical translation.

We also summarize the broader clinical potential of chemokine modulation in cancer intervention. Future research should prioritize precise targeting strategies informed by patient stratification based on chemokine profiles. Promising avenues include nanomaterial-, hydrogel-, or exosome-mediated delivery of chemokine antagonists. Combining these approaches with emerging therapies (e.g., CAR-T cells, photodynamic therapy) may help overcome resistance mechanisms.

Despite the comprehensive summary of current research in this review, several limitations should be acknowledged. First, the conclusions are drawn from a vast and heterogeneous body of literature, encompassing studies that utilized diverse experimental models (e.g., various cell lines, animal models), methodologies, and patient cohorts. This heterogeneity may limit the generalizability of some findings. Second, there exists a significant research imbalance within the chemokine field itself. While CC and CXC subfamilies are relatively well-studied, the functions of other subfamilies (e.g., XC, CX3C) in OSCC remain substantially underexplored, creating a knowledge gap. Furthermore, the dualistic and highly context-dependent nature of many chemokines poses a challenge for drawing definitive, universally applicable conclusions, as their effects can vary dramatically depending on the cellular source, tumor stage, and overall composition of the TME. Finally, while we discuss promising therapeutic strategies, it must be emphasized that the clinical translation of most findings targeting chemokines in OSCC is still in its infancy, with limited robust clinical trial data to support widespread application.

In conclusion, further investigation into chemokine/chemokine receptor interactions in OSCC, coupled with developing targeted therapeutics to disrupt malignant progression and improve prognosis, constitutes a highly promising research direction.
